# Expression of Nutrient Transporters on NK Cells During Murine Cytomegalovirus Infection Is MyD88-Dependent

**DOI:** 10.3389/fimmu.2021.654225

**Published:** 2021-05-03

**Authors:** Abrar Ul Haq Khan, Saeedah Musaed Almutairi, Alaa Kassim Ali, Rosalba Salcedo, C. Andrew Stewart, Lisheng Wang, Seung-Hwan Lee

**Affiliations:** ^1^ Department of Biochemistry, Microbiology, and Immunology, Faculty of Medicine, University of Ottawa, Ottawa, ON, Canada; ^2^ Botany and Microbiology Department, College of Sciences, King Saud University, Riyadh, Saudi Arabia; ^3^ Cancer and Inflammation Program, Center for Cancer Research, National Cancer Institute, National Institute of Health, Bethesda, MD, United States; ^4^ The University of Ottawa Centre for Infection, Immunity, and Inflammation, Ottawa, ON, Canada

**Keywords:** natural killer cells, nutrient transporters, cytokines, MyD88, Ly49H, MCMV: murine CMV

## Abstract

Natural killer (NK) cells are the predominant innate lymphocytes that provide early defense against infections. In the inflammatory milieu, NK cells modify their metabolism to support high energy demands required for their proliferation, activation, and functional plasticity. This metabolic reprogramming is usually accompanied by the upregulation of nutrient transporter expression on the cell surface, leading to increased nutrient uptake required for intense proliferation. The interleukin-1 family members of inflammatory cytokines are critical in activating NK cells during infection; however, their underlying mechanism in NK cell metabolism is not fully elucidated. Previously, we have shown that IL-18 upregulates the expression of solute carrier transmembrane proteins and thereby induces a robust metabolic boost in NK cells. Unexpectedly, we found that IL-18 signaling is dispensable during viral infection *in vivo*, while the upregulation of nutrient transporters is primarily MyD88-dependent. NK cells from *Myd88^-/-^* mice displayed significantly reduced surface expression of nutrient receptors and mTOR activity during MCMV infection. We also identified that IL-33, another cytokine employing MyD88 signaling, induces the expression of nutrient transporters but requires a pre-exposure to IL-12. Moreover, signaling through the NK cell activating receptor, Ly49H, can also promote the expression of nutrient transporters. Collectively, our findings revealed multiple pathways that can induce the expression of nutrient transporters on NK cells while highlighting the imperative role of MyD88 in NK cell metabolism during infection.

## Introduction

Natural killer (NK) cells are predominant effector lymphocytes involved in anti-tumor and anti-viral immune responses and provide the first line of defense without prior sensitization (1, 2). The role of NK cell metabolism in facilitating robust effector functions is emerging as a key aspect in recent studies (3, 4). At a steady-state, NK cells exhibit a metabolically quiescent phenotype that is sufficient to meet their basal energy needs as well as their biosynthetic demands upon activation by short-term cytokine stimulation or activating receptor ligation (5). However, during inflammation, naïve NK cells undergo rapid metabolic reprogramming to support their expansion and differentiation into potent effector cells (6–8). NK cells upregulate numerous solute carrier (SLC) transporters, which mediate several essential physiological functions including nutrient uptake, ion influx/efflux, and waste disposal (9). The most widely investigated nutrient transporters on immune cells are CD71, the transferrin receptor and CD98 (SLC3A2), a chaperone that forms a heterodimeric system L-amino acid transporter by pairing mainly with SLC7A5 (LAT-1) in NK cells (3, 6, 7, 10–12). Activation of NK cells leads to increased glycolysis, highlighted by enhanced mTOR activity and the expression of glycolytic enzymes. Interestingly, NK cell activation also increases the mitochondrial mass to support higher oxidative phosphorylation rates (OXPHOS) (13, 14).

NK cells are activated upon stimulation with cytokines or through the engagement of their various activating receptors (15, 16). Several cytokines are known to modulate NK cell proliferation and effector functions during infection and inflammation (17–19). Among these, interleukin-18 (IL-18), a member of the IL-1 family of cytokines, was initially discovered for its ability to induce IFN-γ production (20–22). This cytokine is mainly produced by macrophages and dendritic cells, and during inflammasome activation, IL-18 is processed from an inactive precursor into an active form by caspase 1-mediated cleavage (21, 23, 24). Signaling through IL-18 is mediated by binding to IL-18 receptor (IL-18R), a heterodimeric complex, which consists of the ligand-binding chain, IL-18Rα, and the signal-transducing chain IL-18Rβ (21, 25, 26). IL-18R is generally expressed in hematopoietic cells, with the highest expression in NK cells (10, 27). Notably, signaling through IL-18 recruits the adapter protein myeloid differentiation primary response gene 88 (MyD88) and leads to the activation of nuclear factor-κB (NF-κB) (17, 28–30). The role of IL-18 in regulating NK cell function and proliferation is well established (10, 31, 32). IL-18 triggers gene transcription and protein synthesis leading to NK cell activation, enhanced proliferation, and effector functions (32, 33). Moreover, IL-18 priming upregulates the expression of IL‐2Rα in NK cells, thereby increasing NK cell sensitivity to IL‐2 (32, 34). Importantly, IL-18 has been shown to support the selective expansion of the Ly49H+ subset of NK cells during murine cytomegalovirus (MCMV) infection (31). In addition, several biological functions of IL-18, including NK cell proliferation and IFN-γ production, have been described in combination with IL-12 (23, 28, 34).

IL-33, identified as a ligand for Interleukin 1 receptor-like 1 (IL1RL1, also known as ST2), stimulates and mediates inflammatory responses following infection and inflammation (35, 36). In the steady-state, various tissues constitutively express IL-33, however, its expression surges during inflammation following its release into the extracellular space. Thus, IL-33 functions as an endogenous risk signal that alerts the immune system (37, 38). The receptor for IL-33 exists in two main splice variants with opposite roles: i) a membrane-bound form (ST2), which can activate the MyD88/NF-κB signaling pathway, and ii) a soluble form (sST2), which acts as a decoy receptor (39). Accumulating evidence suggests that IL-33 potently stimulates group 2 innate lymphoid cells (ILC2) as well as NK cells (35, 36). Notably, the synergistic effects of IL-12 and IL-33 have been described to promote NK cell effector functions (38–41). Mechanistically, IL-12 upregulates the expression of ST2 on NK cells and is pivotal in IL-33-induced NK cell activation (42, 43). Moreover, IL-33 has been shown to augment NK cell activation, proliferation, and IFN-γ production in a MyD88-dependent manner (44–46).

While pro-inflammatory cytokines milieu is critical in regulating NK cell response, NK cell effector functions are also governed by activating and inhibitory signals emanated from a broad array of activating and inhibitory receptors that operate in an overlapping fashion (15, 47). Among the various activating receptors expressed by NK cells, Ly49H is well-defined and is stimulated by the viral glycoprotein m157 during MCMV infection (48–51). Notably, the Ly49H^+^ NK cell subset exhibits heightened effector functions and elevated proliferation rates to constitute the bulk NK cell population during MCMV infection (52–55).

Previously, we have described that the expression of nutrient transporters on NK cells is mediated by IL-18 and thereby dramatically increases NK cell proliferation (10). However, it remains to be determined how the expression of nutrient transporters on NK cells is regulated during viral infection. Herein, we show that, unexpectedly, IL-18 signaling is dispensable during MCMV infection *in vivo.* Instead, we demonstrate that the upregulation of nutrient transporters as well as mTOR activity in NK cells is primarily regulated in a MyD88*-*dependent manner. We further identified that IL-33 signaling induces the expression of nutrient transporters on NK cells but requires the sequential intervention of IL-12. Moreover, we established that signaling through the NK cell activating receptor, Ly49H, also contributes to the expression of nutrient receptors. Altogether, our findings demonstrate that the expression of nutrient transports on NK cells is regulated by multiple pathways during infection *in vivo*.

## Materials and Methods

### Mice

Wild-type C57BL/6 and *Il18r1^-/-^* mice were purchased from The Jackson Laboratory. The *NKp46iCre* knock-in mouse was a generous gift from Dr. Eric Vivier (Centre d’Immunologie de Marseille-Luminy, Marseille, France). This knock-in mouse was generated by homologous recombination in which iCre (improved Cre) was inserted at the 3’ end of the *Nkp46* gene (56). *Il18r1* floxed mice were a kind gift from Dr. Giorgio Trinchieri (Cancer and Inflammation Program, Center for Cancer Research, National Cancer Institute, NIH, Frederick, MD, USA) and were generated by R.S. and C.A.S (57). *MyD88^-/-^* and *Caspase1^-/-^* mice were kindly provided by Dr. Subash Sad at the University of Ottawa. The mice were housed in a specific pathogen-free environment. All mice used for experiments were aged between 6 to 12 weeks old. All procedures were approved by and conducted in accordance with the institution’s animal guidelines of the University of Ottawa.

### MCMV Infection, LPS and IL-18 Injection

Smith strain MCMV stocks were generated in our laboratory from the salivary glands of infected BALB/c mice. Mice were infected with 3,000 PFUs MCMV intraperitoneally. To investigate the role of Ly49H in mice, C57BL/6 mice were challenged with 15,000 PFUs MCMV. To investigate the effect of inflammation on the expression of nutrient transporters, mice were injected intraperitoneally with 50 ug of LPS in PBS. To investigate the role of IL-18 cytokine on NK cell function *in vivo*, mice were injected intraperitoneally with 100 ng of LPS and 100 ng of IL-18 in PBS. To measure BrdU incorporation *in vivo*, mice were injected intraperitoneally with 2 mg of BrdU 2 hours prior to sacrifice.

### Cell isolation, NK Cells Enrichment and *Ex Vivo* Stimulation

Spleens were harvested, and a single-cell suspension was generated following red blood cells lysis and filtration through a 70-µm filter. NK cells were enriched from the spleen by negative selection using the MagniSort Mouse NK cell Enrichment Kit (eBioscience). Purified NK cells were cultured for either 24 or 48 hours with the following cytokines at the indicated concentrations; IL-12 (20 ng/ml), IL-18 (50 ng/ml), and IL-33 (30 ng/ml). 100 U/ml of recombinant human IL-2 (obtained from NCI Preclinical Repository) was added to support NK cell survival. NK cells were cultured in RP-10 media (RPMI-1640 medium containing 10% FBS, penicillin/streptomycin, 2 mM L-glutamine, 10 mmol HEPES, 50 µmol 2-mercaptoethanol). Enriched NK cells were co-cultured with BAF3 or BAF3-m157 cells (gifts from Dr. Wayne Yokoyama, Washington University) at an E:T ratio of 2:1 for 18 hours in the presence of 5 ng/mL IL-15/IL-15Rα complex or 1000U/mL IL-2. Serum levels of IL-18 cytokine were measured using IL-18 Mouse ProcartaPlex™ Simplex Kit (Invitrogen) and acquired using MAGPIX System (Luminex Corporation).

### MCMV Titer Determination

For measuring the viral titers, spleen and liver from infected mice were homogenized by MagNALyser (Roche Applied Science), and the lysates were diluted and overlaid on mouse embryonic fibroblasts cells for 1 h at 37°C in 2% DMEM (DMEM medium supplemented with 2% FBS, 1× penicillin/streptomycin, 2 mM L-glutamine, 10 mmol HEPES, and 50 μmol 2-mercaptoethanol). After 1 hour incubation, the virus was removed from the monolayers by aspiration. The monolayers were overlaid with 1 part of DMEM containing 2% low melting agar mixed with 3 parts of 13.5% DMEM (DMEM medium supplemented with 13.5% FBS, 1× penicillin/streptomycin, 2 mM L-glutamine, 10 mmol HEPES, and 50 μmol 2-mercaptoethanol). Three days later, the cells were fixed with 10% formalin for 10 min and stained with 1% Crystal Violet for 10 min. Plaques were counted and represented as log PFU/g of organs.

### Antibodies and Flow Cytometry

The following mAbs were used: anti-CD3 (17A2 and 145-2C11), anti-TCRβ (H57-597), anti-CD8 (53-6.7), anti-CD49b (DX5), anti-phospho-S6 (Ser-235/Ser-236), and anti-IL-18Rα (P3TUNYA) from eBioscience; anti-CD19 (1D3), anti-CD4 (RM4-5), anti-F4/80 (T45-2342), anti-NK1.1 (PK136), anti-Ki-67 (B56), and anti-BrdU (3D4) from BD Biosciences; anti-CD71 (RT7217), anti-CD98 (RL388) from Biolegend; and Live/Dead Fixable Yellow Dead Cell Stain from Invitrogen. Intracellular staining of Ki-67 was carried out using a Foxp3 staining kit (eBioscience). Mitochondrial mass was measured using nonylacridine orange (NAO) (Thermo Fisher Scientific). Cells were acquired using BD LSRFortessa or Thermofisher Attune NxT and analyzed using Kaluza 1.3 Analysis software (Beckman Coulter) or FlowJo (V10).

### Glucose Uptake Assay

5×10^5^ to 10^6^ spleen cells/ml were washed with PBS and incubated for 15 min in RPMI-1640 without glucose (Corning) supplemented with 10% of dialyzed serum (Thermo Fisher Scientific), 2 mM L-glutamine, 1 mM HEPES, 1% penicillin/streptomycin and 50 µmol 2-mercaptoethanol at 37°C. Cells were incubated for 1 hour in the glucose-free medium with 50 µM of 2-NBDG (Life Technologies) at 37°C. Cells were washed twice with PBS and stained for NK1.1, TCRβ, and Fixable Yellow Live/Dead (Invitrogen) on ice for 25 min, before being analyzed using flow cytometry.

### OCR Measurement by Seahorse

Isolated NK cells were cultured for 7–10 days in the presence of 1,000 units/ml recombinant human IL-2 and treated with or without 30 ng/ml IL-18 for 24 hours. XF 24-well microplates (Seahorse Bioscience) were precoated with Cell-Tak (Corning) for 2 hours before seeding the NK cells on the plate for real-time analysis of the Oxygen consumption rate (OCR). 10^6^ NK cells were cultured per well, and various inhibitors were added (Agilent Seahorse XF Cell Mito Stress Test) at the following concentrations: oligomycin (2 μm), FCCP (0.5 μm), and rotenone (100 nM) plus antimycin A (4 μM), which allow the accurate calculation of respiration capacity (OCR).

### Statistical Analysis

The mean values in the experiment were tested by ANOVA. If the ANOVA rejected the null hypothesis of the same means among the conditions (*p* < 0.01), multiple comparisons were performed between selected pairs of means by two-tailed unpaired t-test (*p < 0.05, **p < 0.01, ***p < 0.001), using Prism Version 8 (GraphPad Software).

## Results

### Augmented Proliferation of NK Cells Correlates With Higher Expression of Nutrient Receptors During MCMV Infection

**Figure 1:**
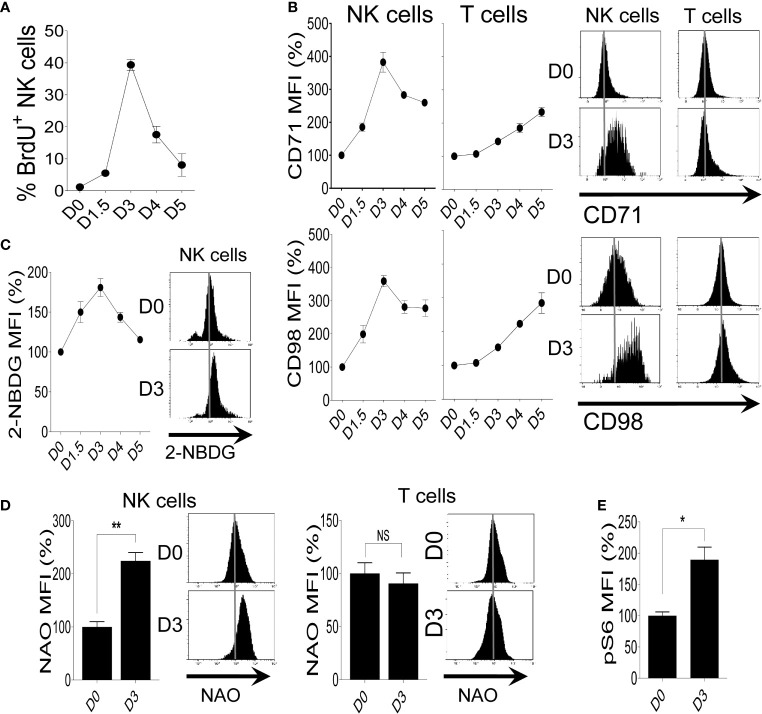
Increased proliferation of NK cells correlates with higher expression of nutrient receptors during MCMV infection. C57BL/6 mice were either left untreated or infected with 3,000 PFU MCMV intraperitoneally and splenocytes were analyzed on the indicated days. **(A)** Kinetics of NK cell proliferation during MCMV infection as measured by BrdU incorporation. **(B)** Representative plots depict the mean fluorescence intensity (MFI) of CD71 and CD98 expression on NK cells and T cells in the spleens of naive (D0) or MCMV-infected mice at day 3 post-infection (D3). **(C)** Representative plots of the glucose uptake by NK cells during MCMV infection as measured by the MFI of 2-NBDG. **(D)** Representative plots depict the MFI of NAO expression on NK cells and T cells in the spleens of naive (D0) or MCMV-infected mice at D3. **(E)** Representative plots depict the MFI of pS6 expression on NK cells in the spleens of naive (D0) or MCMV-infected mice at D3. The MFI expression is presented in percentage relative to the MFI of control mice as 100. Data are from one experiment representative of two independent experiments, with three to five mice per group. Data represent mean + SD. NS, non-significant; *p < 0.05; **p < 0.01.

To investigate the metabolic activity of NK cells during MCMV infection, we first measured NK cell proliferation *via* BrdU incorporation. NK cells from C57BL/6 mice undergo enhanced proliferation, reaching a peak on day 3 (D3) post-infection (**Figure 1A**). Proliferating NK cells demand high energy required for the synthesis of diverse cellular compartments. We reasoned that the elevated metabolic demand of NK cells as a result of their augmented proliferation can be fulfilled by increasing the expression of nutrient transporters to allow enhanced nutrient uptake. To investigate whether the expression of nutrient transporters also corresponds to NK cell proliferation following MCMV infection, we analyzed the expression of transferrin receptor (CD71) and amino acid transporter (CD98) on NK and T cells from infected mice. NK cells displayed enhanced expression of nutrient transporters, reaching a peak on D3 post-infection compared to basal expression at D0 (**Figure 1B**). There was a negligible increase in the expression of CD71 and CD98 on T cells during the early stage of infection. Activated NK cells have a substantial increase in both glycolysis and OXPHOS (3), and glucose is a critical fuel that drives glycolysis as well as OXPHOS in NK cell for proliferation and effector function (13, 58). To evaluate the expression of glucose transporters, we next analyzed the glucose uptake capacity of NK cells after MCMV infection by using 2-NBDG, a fluorescent glucose analog. We observed higher 2-NBDG uptake in NK cells and peaks at D3 post-infection, suggesting enhanced expression of glucose transporters, consistent with their proliferation rate (**Figure 1C**). Taken together, proliferating NK cells during MCMV infection express higher expression of nutrient transporters.

We have previously demonstrated that *ex vivo* stimulation of naïve NK cells with IL-18 upregulated the expression of CD71 and CD98 (10). Thus, we investigated whether IL-18 can induce NK cell proliferation and increase the expression of nutrient receptors *in vivo*. To examine this mice were injected with IL-18 and NK cells were analyzed at D2 post-injection. Mice were also injected with LPS as it has been shown to enhance NK cell proliferation and the expression of nutrient receptors (10, 59). Notably, NK cells from mice injected with IL-18 have a significantly higher proliferation rate as measured by BrdU incorporation (**Figure S1A**) and expression of proliferation antigen Ki-67 (**Figure S1B**). Notably, administration of IL-18 in combination with LPS injection further augmented the proliferation of NK cells (**Figures S1A, B**). This increase in NK proliferation accompanied by the higher expression of nutrient transporters in a consequent fashion (**Figure S1C**) suggesting a sturdy association between proliferation and the expression of nutrient transporters.

Mitochondria are key metabolic organelles that regulate cellular growth through efficient ATP production, and higher mitochondrial mass is an indication of superior transcription and translation rates in proliferating cells (60). To validate this, freshly isolated NK cells were stimulated with different concentrations of IL-2 and mitochondrial mass was determined by nonyl acridine orange (NAO), a dye that binds to mitochondrial proteins independently of membrane potential (61). We observed a dose-dependent upregulation of mitochondrial mass in IL-2 stimulated NK cells (**Figure S2A**). As expected, IL-18 stimulation also resulted in higher mitochondrial mass of NK cells (**Figure S2B**). Previously, we showed that IL-18 stimulation leads to an increased glycolysis rate (10). Here, we determined if this increased mitochondrial mass supports a high rate of OXPHOS. Following IL-18 stimulation, higher mitochondrial mass in NK cells is accompanied by elevated OXPHOS and maximal respiration rate as measured by oxygen consumption rate (**Figure S2C**). Next, we measured the mitochondrial mass after MCMV infection. Consistent with the proliferation and the expression of nutrient receptors, NK cells at D3 showed a higher mitochondrial mass, whereas this observation was not made in T cells (**Figure 1D**). Lastly, NK cells from MCMV infected mice also displayed higher expression of mTOR activity, a master regulator of NK cell metabolism (3), as measured by pS6 (**Figure 1E**). Overall, these results demonstrate that following MCMV infection or LPS-induced inflammation, the robust proliferation and the elevated expression of nutrient transporters on NK cells are accompanied by higher metabolic activity.

### Generation of NK Cell-Specific *Il18r1*-Deficient (*NKp46-Cre-Il18r1^fl/fl^*) Mice

**Figure 2:**
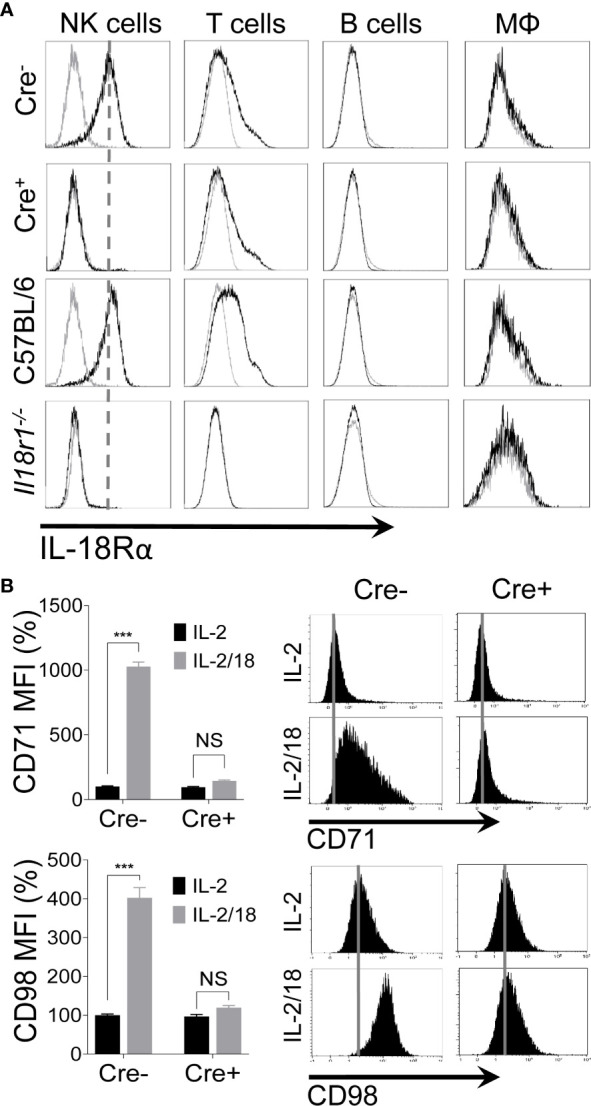
Generation of NK cell-specific IL-18 receptor-deficient mice. Excision of *Il18r1* allele occurs predominantly in NK cells of *NKp46-Cre-Il18r1^fl^* ^/^ *^fl^* mice. **(A)** Protein expression of the IL-18 receptor alpha (IL-18Rα) on different subsets of splenic leukocytes from indicated mice. **(B)** NK cells from spleens of naïve *Il18r1^fl^* ^/^ *^fl^* (Cre-) and *NKp46-Cre-Il18r1^fl^* ^/^ *^fl^* (Cre+) littermate mice were enriched and stimulated with IL-18 *ex vivo* for 24 hours. 100 U/ml of rhIL-2 was added to maintain NK cell survival. Representative plots depict the mean fluorescence intensity (MFI) of CD71 and CD98 expression on cytokine-stimulated NK cells. The MFI expression is presented in percentage relative to the MFI of unstimulated cells as 100. Data are from one experiment representative of 3 independent experiments, with at least two replicates per group. Data represent mean + SD. NS, non-significant; ***p < 0.001.

The observation that IL-18 upregulates the expression of nutrient transporters on NK cells (10) prompted us to assess this pathway *in vivo*. To investigate the role of IL-18 signaling *in vivo*, we generated a mouse model that lacks the *Il18r1* gene specifically in NK cells (*NKp46-Cre-Il18r1^fl/fl^*) by crossing an *NKp46iCre* knock-in mouse (56) with an *Il18r1* floxed mouse (*Il18r1^fl/fl^*) (57). To validate that Cre-mediated recombination occurs exclusively in NK cells, we analyzed the expression of interleukin-18 receptor alpha (IL-18Rα) on NK cells, T cells, B cells, and macrophages from splenocytes of *Il18r1^fl/fl^* (Cre-) and *NKp46-Cre-Il18r1^fl/fl^* (Cre+) littermate mice. The expression of IL-18Rα was absent on the surface of NK cells from Cre+ mice as measured by flow cytometry (**Figure 2A**). Notably, the expression of IL-18Rα remained unchanged on T cells indicating that Cre-mediated recombination occurred exclusively in NK cells and not in other cells (**Figure 2A**). The Cre+ mice were fertile and obtained at Mendelian frequency. IL-18Rα expression in Cre- corresponded to that in C57BL/6 mice, while its expression in the whole body *Il18r1* ^-/-^ mice was absent in all cell types. Thus, Cre-mediated recombination in *NKp46-Cre-Il18r1^fl/fl^* mice resulted in *Il18r1* deletion specifically in NK cells without affecting the phenotype of other major lymphocyte populations.

To confirm that IL-18 signaling was abrogated in the absence of IL-18Rα expression in our mouse model, we stimulated freshly isolated NK cells from Cre- and Cre+ mice with IL-18 *ex vivo*. As expected, IL-18 stimulation upregulated the expression of CD71 and CD98 on NK cells only in Cre- mice, whereas NK cells from Cre+ mice failed to upregulate the expression of nutrient transporters (**Figure 2B**). Comparable results were observed when NK cells from C57BL/6 and *Il18r1* ^-/-^ mice were stimulated with IL-18 *ex vivo.* NK cells from wild-type mice responded to stimulation, resulting in the upregulated expression of CD71 and CD98 on NK cells, while this effect was absent in NK cells from *Il18r1* ^-/-^ mice (**Figure S3**). Taken together, we generated NK cell-specific *Il18r1*-deficient mice as a model to study the role of IL-18 signaling in the upregulation of nutrient transporters on NK cells *in vivo*.

### IL-18 Signaling Is Dispensable During MCMV Infection

**Figure 3:**
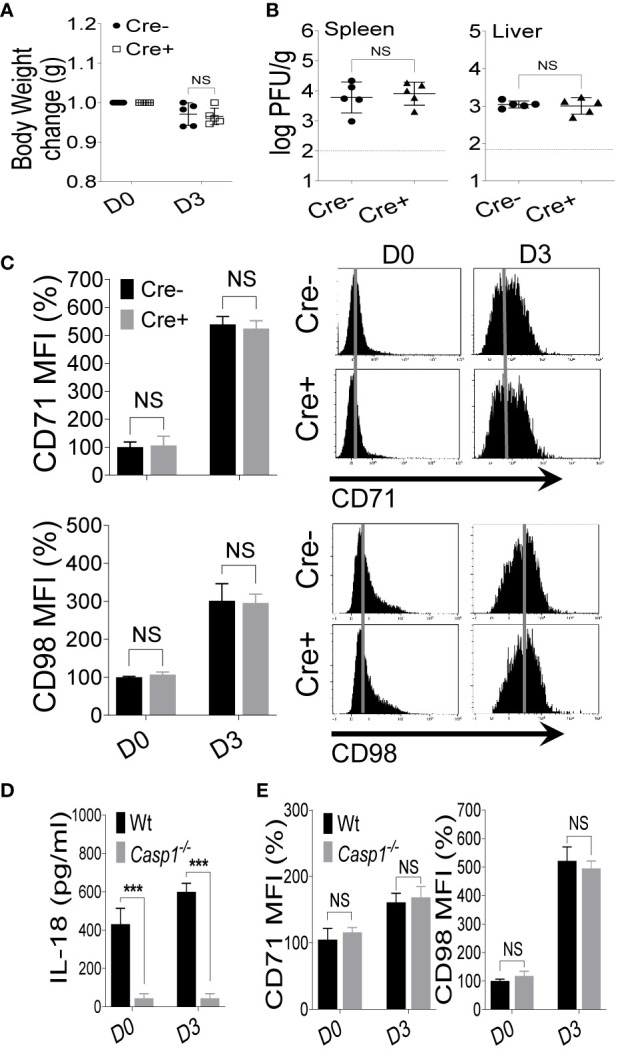
IL-18 signaling is dispensable during MCMV infection. *Il18r1^fl/fl^* (Cre*-*) and *NKp46-Cre-IL18r1^fl/fl^* (Cre+) mice were either left untreated or infected with 3,000 PFU MCMV intraperitoneally and analyzed on the indicated day. **(A)** Bodyweights change of infected mice relative to their initial body weight at day 0 (D0). **(B)** Viral titers in the spleen and liver of *Il18r1^fl^* ^/^ *^fl^* and *NKp46-Cre-Il18r1^fl^* ^/^ *^fl^* mice at day 3 (D3) post-infection. **(C)** Representative plots depict the mean fluorescence intensity (MFI) of CD71 and CD98 expression on NK cells in the spleens of naive (D0) or MCMV-infected mice at D3. **(D)** C57BL/6 and *Caspase1^-/-^* mice were either left untreated or infected with 3,000 PFU MCMV intraperitoneally and analyzed on the indicated day. Serum levels of IL-18 were measured at D3 post-infection. **(E)** Representative plots depict the MFI of CD71 and CD98 expression on NK cells in the spleens of naive (D0) or MCMV-infected mice at D3 post-infection. The MFI expression is presented in percentage relative to the MFI of untreated mice as 100. Data are from one experiment representative of 3 independent experiments with three to five mice per group. Data represent mean + SD. NS, non-significant; ***p < 0.001.

We have demonstrated that the expression of CD71 and CD98 on NK cells is upregulated following IL-18 stimulation (**Figure 2B**) (10). To determine whether IL-18 signaling is necessary for the expression of nutrient transporters *in vivo* during viral infection, Cre- and Cre+ mice were infected with MCMV, and NK cells in the spleens of these mice were analyzed for the expression of nutrient transporters at D3 post-infection. Cre+ mice showed comparable body weight losses to those of Cre- mice (**Figure 3A**). Moreover, viral loads in the spleen and liver of infected mice were similar between the two groups (**Figures 3B** and **S4A**). Unexpectedly, the expression of CD71 and CD98 on NK cells from Cre+ mice were comparable to those of NK cells from Cre- mice (**Figure 3C**). Consistent with these results, no difference was observed when comparing C57BL/6 and the whole body *Il18r1* ^-/-^ mice upon challenge with MCMV (**Figure S4B**). Previous results indicated that following LPS injection, NK cells upregulate the expression of nutrient transporters (10). We injected C57BL/6 and *Il18r1* ^-/-^ mice with LPS and analyzed the expression of CD71 and CD98 on NK cells on D2 post-injection. Once again, we did not detect any noticeable difference in the expression of these receptors between wild-type and *Il18r1* ^-/-^ mice post LPS infection (**Figure S4C**), indicating that NK cells can upregulate the expression of nutrient transporters in the absence of IL-18 signaling during *in vivo* inflammation.

IL-18 is processed from an inactive precursor into an active form through a caspase 1-mediated cleavage (21, 24). To further illustrate the role of the IL-18 cytokine in the upregulation of nutrient transporters on NK cells, we infected wild-type and *Caspase1^-/-^* mice with MCMV. As expected, *Caspase1^-/-^* mice failed to produce functional IL-18 based on the low concentrations of IL-18 in the serum (**Figure 3D**). However, consistent with data from *Il18r1*-deficient mice, we did not observe any differences in regard to the expression levels of CD71 and CD98 on NK cells at D3 post-MCMV infection (**Figure 3E**). Taken together, these results demonstrate that IL-18 signaling is dispensable for the upregulation of nutrient transporters on NK cells during *in vivo* infection and inflammation.

### Upregulation of Nutrient Transporters Expression on NK Cells During MCMV Infection Is Myd88-Dependent

The binding of IL-18 to its receptors triggers an orchestrated signaling pathway that begins with the recruitment of the cytoplasmic adaptor molecule, MyD88, and leads to the activation of NF-κB (62). Thus, we investigated whether the upregulation of nutrient receptors on NK cells is MyD88-dependent. First, freshly isolated NK cells from C57BL/6 and *MyD88^-/-^* mice were subjected to *ex vivo* stimulation with IL-12 and IL-18. NK cells from *MyD88^-/-^* mice failed to upregulate the expression of CD71 and CD98 (**Figure S5A**), suggesting that IL-18-induced upregulation of nutrient transporters requires the intrinsic MyD88 pathway.

**Figure 4:**
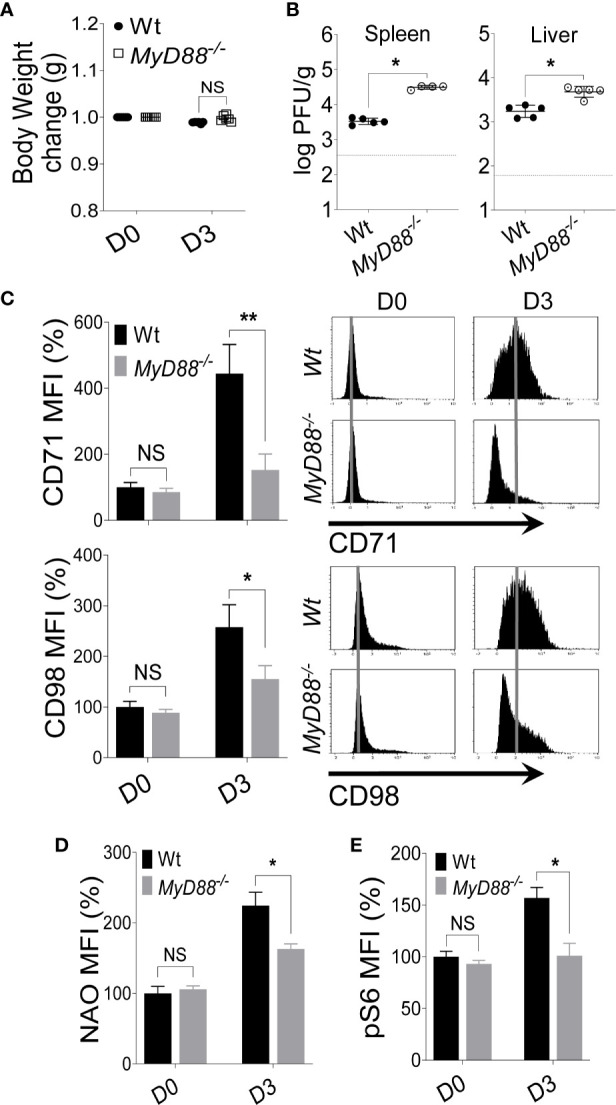
Upregulation of nutrient transporters on NK cells during MCMV infection is MyD88 dependent. C57BL/6 and *MyD88^-/-^* mice were either left untreated or infected with 3,000 PFU MCMV intraperitoneally and analyzed on the indicated day. **(A)** Bodyweight change of infected mice relative to their initial body weight at day 0 (D0). **(B)** Viral titer in the spleen and liver of C57BL/6 and *MyD88^-/-^* mice at day 3 (D3) post-infection. **(C)** Representative plots depict the mean fluorescence intensity (MFI) of CD71 and CD98 expression on NK cells in the spleens of naive (D0) or MCMV-infected mice at D3 post-infection. Representative plots depict the MFI of **(D)** NAO expression and, **(E)** pS6 expression on NK cells in the spleens of naive (D0) or MCMV-infected mice at D3 post-infection. The MFI expression is presented in percentage relative to the MFI of untreated mice as 100. Data are from one experiment representative of 3 independent experiments with three to five mice per group. Data represent mean + SD. NS, non-significant, *p < 0.05; **p < 0.01.

Next, we infected C57BL/6 and *MyD88^-/-^* mice with MCMV and examined the expression of nutrient transporters on NK cells at D3 post-infection. No difference in body weight change was observed between C57BL/6 and *MyD88^-/-^* mice after MCMV infection (**Figure 4A**), however *MyD88^-/-^* mice showed a higher viral load in the spleen and liver (**Figure 4B**). Notably, NK cells from *MyD88^-/-^* mice showed significantly reduced expression of CD71 and CD98 compared to their wild-type counterparts (**Figure 4C**). Although the increase in CD71 expression on NK cells from *MyD88^-/-^* mice at D3 was negligible, we did still observe a slight increase in the expression of CD98 (**Figure 4C**), suggesting that CD98 expression is regulated by a MyD88-independent mechanism. To assess the metabolic activity, we measured mitochondria mass and mTORC1 activity. The decreased expression of nutrient receptors was accompanied by lower NAO staining (**Figure 4D**) and pS6 (**Figure 4E**) in *MyD88^-/-^* mice compared to wild-type mice at D3 post-MCMV infection. Similar results were obtained when the mice were injected with LPS. At D2 post-injection, NK cells from *MyD88^-/-^* mice expressed considerably low levels of CD71 and CD98 (**Figure S5B**). Collectively, our data demonstrate that signaling through MyD88 is imperative in upregulating the expression of nutrient receptors and metabolic activity of NK cells.

### IL-33 Signaling Upregulates the Expression of Nutrient Transporters in Combination With IL-12

**Figure 5:**
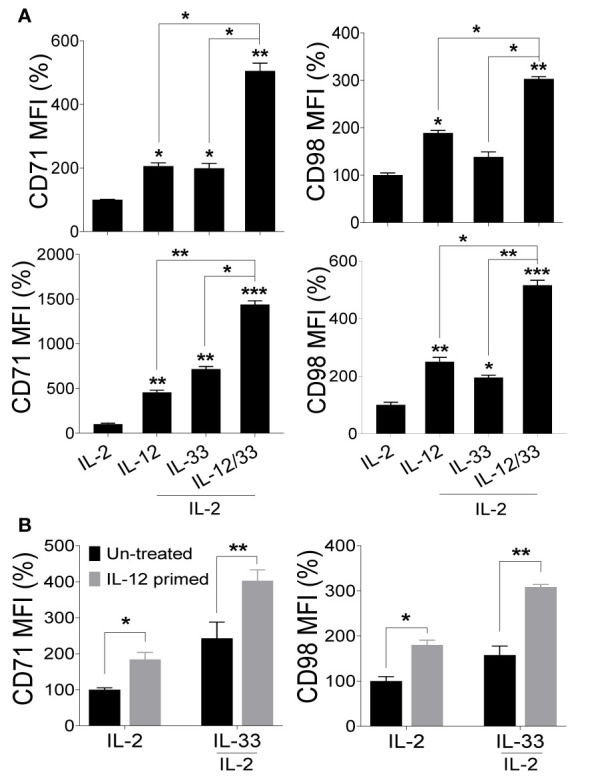
Signaling through IL-33 upregulates nutrient transporters on NK cells but requires a sequential intervention of IL-12. NK cells were enriched from the spleens of naive C57BL/6 mice and stimulated with the indicated cytokines *ex vivo*. 100 U/ml of rhIL-2 was added to maintain NK cell survival. **(A)** Representative graphs depict the mean fluorescence intensity (MFI) of CD71 and CD98 expression on cytokine-stimulated NK cells after 24 hours (upper panel) and 48 hours (lower panel). **(B)** NK cells from splenocytes of C57BL/6 mice were enriched and either left untreated (black bars) or primed with IL-12 (grey bars) for 48 hours followed by complete removal of media and re-stimulation with IL-33 for 24 hours. 100 U/ml of rhIL-2 was added to maintain NK cell survival. Representative graphs depict the MFI of CD71 and CD98 expression on NK cells. The MFI expression is presented in percentage relative to the MFI of unstimulated cells as 100. Data are from one experiment representative of 3 independent experiments, with at least two replicates per group. Statistics are comparing samples to IL-2 stimulated NK cells. Data represent mean + SD. *p < 0.05; **p < 0.01; ***p < 0.001.

Given the observation that IL-18 signaling is dispensable during MCMV infection while the expression of nutrient transporters on NK cells *in vivo* requires the MyD88 pathway, we reasoned that IL-18-induced upregulation of nutrient transporters is compensated by cytokines that share the MyD88 pathway. Among the members of the IL-1 family cytokines that require the MyD88 pathway for signaling, IL-33 has been shown to regulate NK cell proliferation and activation (41, 43). Interestingly, most of the effects of IL-33 in promoting NK cell effector functions have been described in combination with IL-12 (38–41). Thus, we investigated whether IL-33 alone or in combination with IL-12 can upregulate the expression of CD71 and CD98 on NK cells. Notably, we detected a modest increase in the expression of nutrient transporters on NK cells after 24 hours of *ex vivo* stimulation with IL-12 or IL-33 alone, however, the combination of both cytokines greatly induced the expression of these transporters (**Figure 5A**, upper panel), demonstrating that these cytokines have synergistic effects. Strikingly, prolonged stimulations up to 48 hours further heightened the expression of both CD71 and CD98 (**Figure 5A**, lower panel).

IL-12 was previously shown to upregulate the expression of ST2, rendering NK cells sensitive to IL-33 action (42, 43). To determine whether IL-12 primes NK cells for IL-33-mediated upregulation of nutrient transporters, freshly isolated NK cells from naïve C57BL/6 mice were either left untreated (black bars) or pre-stimulated with IL-12 (gray bars) for 48 hours followed by stimulation with IL-33 for 24 hours. Although NK cells pre-treated with IL-12 showed a marginal increase in nutrient transporters expression, we noted that IL-12 treatment followed by IL-33 stimulation further improved the expression of CD71 and CD98 (**Figure 5B**), suggesting that NK cells primed with IL-12 further promotes IL-33 signaling. Although IL-12 and IL-33 have synergistic effects on NK cells (**Figure 5A**), these cytokines seem to function in a sequential manner since NK cells become more responsive to IL-33 following pre-exposure to IL-12. Collectively, these data suggest that IL-33 signaling upregulates the expression of nutrient transporters on NK cells but requires priming by IL-12 for optimal signaling.

### Ly49H Signaling Contributes to the Expression of Nutrient Transporters on NK Cells During MCMV Infection

**Figure 6:**
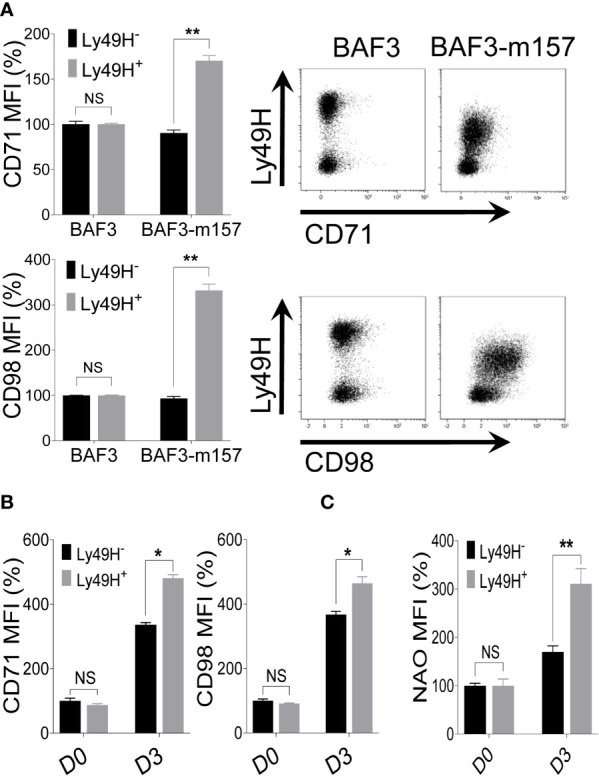
Ly49H signaling is also involved in the regulation of nutrient transporters expression on NK cells upon MCMV infection. **(A)** NK cells were enriched from the spleen of naive C57BL/6 mice and co-cultured with either BAF3 or BAF3-m157 cells for 18 hours. The mean fluorescence intensity (MFI) of CD71 and CD98 expression on NK cells was measured by flow cytometer. Data are from one experiment representative of 2 independent experiments with at least two replicates per group. **(B)** C57BL/6 mice were either left untreated or infected with 15,000 PFU MCMV intraperitoneally and analyzed on the indicated day. Representative plots depict the MFI of CD71 and CD98 expression on NK cells in the spleen of naive (D0) or MCMV-infected mice at day 3 post-infection (D3). **(C)** Representative plots depict the MFI of NAO expression on NK cells in the spleens of naive (D0) or MCMV-infected mice at D3 post-infection. Data are from one experiment representative of 2 independent experiments with three to five mice per group. The MFI expression is presented in percentage relative to the MFI of control as 100. Data represent mean + SD. NS, non-significant, *p < 0.05; **p < 0.01.

The activating receptor Ly49H stochastically expressed on NK cells recognizes the MCMV protein m157 leading to the augmented expansion of MCMV-specific Ly49H^+^ NK cells (50, 52, 53). Since the expansion phase of NK cells during MCMV infection depends mainly on Ly49H engagement, we assessed whether signaling through Ly49H may contribute to NK cell metabolic activity through upregulation of the nutrient transporters. To test this, freshly isolated NK cells from naïve C57BL/6 mice were co-cultured with either parental BAF3 cells or BAF3 cells expressing the m157 MCMV glycoprotein (BAF3-m157) *ex vivo*. Notably, the Ly49H+ NK cells displayed a significantly higher upregulation of CD71 and CD98 expression (**Figure 6A**), and the enhanced expression of nutrient transporters was strictly Ly49H-dependent. This expression was further enhanced in the presence of IL-18, where cells that were stimulated with a relatively low amount of IL-18 displayed an augmented expression of CD71 and CD98 compared to those stimulated with BAF3-m157 cells alone in the absence of IL-18 (**Figure S6**). Next, we examined the expression of CD71 and CD98 on NK cells from MCMV-infected C57BL/6 mice at D3 post-infection in regard to Ly49H expression. Consistent with previous results (**Figure 4C**), the expression of nutrient transporters was upregulated on total NK cells (Ly49H^-^ and Ly49H^+^) at D3, however, the magnitude of this upregulation of both CD71 and CD98 expression was significantly higher in Ly49H^+^ NK cells compared to Ly49H^-^ NK cells (**Figure 6B**). Similarly, Ly49H^+^ NK cells displayed significantly higher mitochondrial mass than Ly49H^-^ NK cells (**Figure 6C**), further supporting the previous data that proliferating cells have higher mitochondrial contents (**Figures S2A, B**). Collectively, these data indicate that signaling through the activating receptor, Ly49H, contributes to the upregulation of nutrient transporters expression on NK cells during MCMV infection.

## Discussion

NK cells are innate lymphocytes with a dominant role in the early defense against tumors and intracellular pathogens, and their dysfunction leads to added susceptibility to virus infections. The acquisition of full effector function in NK cells require substantial metabolic reprogramming (2, 3). Recent research has defined enhanced cellular metabolism in lymphocytes, including NK cells, as indicative of immune responsiveness, while impaired metabolism resulted in immune cell dysfunction and compromised immunity. At the same time, elevated cellular metabolism is a common feature of many activated immune cells to sustain immune functions (3, 4, 63). During activation, NK cells undergo substantial changes in their metabolic pathways to fulfill profound energy demands (7, 63, 64). NK cells upregulate numerous solute carrier transporters to facilitate the uptake of nutrients, and among these transporters, CD71 and CD98 have been extensively studied (6, 9–12). We and others have shown that cell proliferation and metabolic activity correlate in NK cells (6, 10), and proliferating cells exhibit higher metabolic activity and mitochondrial mass (3, 14).

Rapidly dividing and metabolically active cells require a constant supply of nutrients, and glucose along with amino acids are major fuels to feed these cells. However, the influx of the nutrients from the extracellular milieu into cells through the cell membrane requires specific nutrient transporters. Glucose is imported mainly *via* a glucose transporter GLUT1 (known as SLC2A1), and there are 11 SLC families dedicated to transport amino acids (65–67). The expression of these transporters are transcriptionally upregulated in proliferating cells and is primarily regulated by the PI3K-mTOR pathway (65). Silencing through miRNAs have also been shown to regulate the expression of different nutrient transporters at the post-transcriptional level (66). Recently, transcription factor cMyc has been shown to regulate the expression of CD71 in murine NK cells (7), while the expression of CD98 has been shown to be regulated by IL-2 and IL-12/15 on NK cells (11, 68). Interestingly, CD98 can stabilize the expression of GLUT1, resulting in enhanced glucose transport into the cells (69).

Metabolic regulation in NK cells is driven by intricate molecular mechanisms. The mechanistic target of rapamycin (mTOR) is critical for NK cell metabolism and optimal function while decreased mTOR activity in NK cells correlates with reduced metabolism and proliferative potential (6, 10, 70). Importantly, blocking mTORC1 activity significantly diminished the basal expression of CD71 and CD98 on NK cells (71), on the other hand, CD98/LAT1 mediated uptake of L-leucine can drive mTORC1 activation (10), showing a reciprocal interaction of mTOR and expression of nutrient receptors. In fact, expression of CD98 is crucial for cellular homeostasis (72), metabolism (69) and, consequently, proliferation (73). Genetic ablation of CD98 and LAT1 results in reduced clonal expansion of T cells and tumor growth respectively (74, 75) reflecting a critical role of CD98/LAT1 in NK cells metabolism and proliferation.

NK cells preferentially utilize glucose driven glycolysis and OXPHOS to drive NK cell effector functions. This increase in basal OXPHOS is accompanied by higher mitochondrial mass that has also been observed during MCMV infection (13, 14). Mitochondria, also regulated by mTOR signaling, are an important metabolic biomarker and pivotal for OXPHOS activity (76). Glutamine is another important fuel that feeds into the TCA cycle in metabolically active cells, and its availability is essential for NK cell metabolism and effector functions. Moreover, glutamine assists in the uptake of amino acids essential for high rates of cMyc protein synthesis through the L-type amino acid transporter CD98/LAT-1. Withdrawal of glutamine or blockade of amino acid transport leads to loss of cMyc expression and concomitant impairment of NK cell effector functions (7, 77).

We previously reported that IL-18 stimulates NK cell proliferation *via* upregulation of nutrient transporters on NK cells (10). To further investigate the role of IL-18 in NK cell metabolism *in vivo* during infection and inflammation, we generated a mouse model that lacks *Il18r1* specifically in NK cells. We employed MCMV infections, an established model to study NK cell function in response to a viral challenge (31). Surprisingly, we found that IL-18 signaling is dispensable during MCMV infection and blocking IL-18 signaling in mice did not perturb NK cell metabolic activity in regard to their expression of nutrient transporters. Moreover, the viral burden in wild-type and *Il18r1*-deficient mice was comparable. Interestingly, similar observations have been reported indicating the redundant role of IL-18 signaling in the proliferation of Ly49H+ NK cells (78, 79), and its important but not absolute contributory role during MCMV infection *in vivo* (80). Concurrent with our findings, a previous study showed that the local production of IFN-γ in the liver is intact in the absence of IL-18 and is sufficient for host survival upon MCMV infection (81). Taken together, our data support the notion that signaling through IL-18 is not outright for NK cell proliferation, metabolic activity, and NK cell-dependent protection during primary MCMV infection (82).

Signaling through MyD88 is crucial for host defense as the MyD88 pathway is vital not only for Toll-like receptors (TLRs), but is also required by the members of the interleukin-1 cytokine family, including IL-18 and IL-33 (82, 83). Although, the expression of several TLRs on murine NK cells has been suggested, their roles in viral infection have not been clearly demonstrated (84). However, mice deficient in *MyD88* have NK cells with defective IFN-γ production and are susceptible to infections (46, 85, 86). Interestingly, a recent paper showed that IL-18-MyD88 signaling is critical in expanding CD4^+^IFNγ^+^ T cells during parasitic infection. This study also showed the crucial role of MyD88 in CD4^+^ T cell activation, proliferation and protection from apoptosis (83). During MCMV infection, *MyD88^-/-^* mice presented with decreased NK cell function and increased susceptibility mainly due to the defect of pathogen-associated molecular patterns (PAMPs) sensing in dendritic cells leading to reduced production of Type I IFN, IL-12 and TNF-α (80, 85). We found that NK cells from MyD88-deficient mice were unable to upregulate the expression of CD71 and CD98 following infection and these mice exhibited defects in NK cell-mediated viral clearance as displayed by higher viral loads. Thus, we reason that NK cell activation during MCMV infection occurs through cell-intrinsic MyD88 signaling by means of IL-18 and/or IL-33 stimulation and is highly linked to NK cell metabolism by inducing nutrient transporters.

Interestingly, the expression of CD98 was not completely abrogated in *MyD88^-/-^* mice following MCMV infection, suggesting the presence of alternative MyD88-independent pathways for regulating CD98 expression. This is of particular importance that *MyD88^-/-^* mice have the ability to induce the activation of NF-κB and the mitogen-activated protein (MAP) kinase family upon LPS injection through a MyD88-independent mechanism (62, 87). Moreover, several pathways have been demonstrated to regulate the expression of nutrient transporters. For example, IL-2, IL-12/15 have been described to regulate the expression of the nutrient receptors on NK cells (11, 68). Furthermore, the production of residual IL-12 and subsequent IL-12-dependent responses can result in NK cell activation in *MyD88^-/-^* mice (82).

Our work further highlights that signaling through IL-33 induces the expression of nutrients transporters on NK cells. IL-33 is a pleiotropic cytokine of the IL-1 family (35), and most of the function of IL-33 has been shown in combination with IL-12, including the activation of NK cells (43, 88). We observed similar findings and found that a combination of IL-12 and IL-33 triggered a higher expression of CD71 and CD98 on NK cells. Interestingly, IL-33 has been described to augment the proliferation of Ly49H^+^ NK cells during MCMV infection (41). Moreover, NK cells stimulated with a combination of IL-33 and IL-12 *in vitro* displayed strong activation and cytotoxicity (38, 43, 44), indicating the role of IL-33 signaling in NK cells proliferation and function. Similarly, transgenic expression of IL-33 significantly increased the activation, proliferation, and cytotoxicity of NK cells due to enhanced NF-κB signaling and promoted their tumor infiltration *in vivo* (45). Observations from these complementary studies support our findings in an agreement that IL-33 regulates the metabolic activity of NK cells, consequently enhancing their function. However, we also noted that the priming of NK cells with IL-12 augmented the expression of nutrient transporters following IL-33 stimulation. This is consistent with the fact that IL-12 stimulates the expression of ST2 on NK cells, thus rendering NK cells sensitive to IL-33 signaling (42, 43).

Finally, signaling through activating receptors also contributes to NK cell metabolic activity. The recognition of infected and stressed cells by NK cells occurs through multiple germ line-encoded receptor-ligand interactions (15). Despite the pivotal role of cytokines, NK cell function is stringently regulated by the expression of activating and inhibitory cell surface receptors, and engagement of these receptors determines the outcome of NK cell function (50, 52, 53). Notably, NK cell activation *via* receptor ligation is more metabolically dependent compared to cytokine stimulation (5). Moreover, the activating receptor NKG2D regulates the expression of SLC3A2 in a mTORC1 dependent manner (89). Since we and others have observed an intimate correlation of proliferation and metabolic activity, we tested whether the activating receptor Ly49H has a role in NK cell metabolism and found that signaling through Ly49H can also contribute to NK cell metabolic activity. During MCMV infection, Ly49H^+^ NK cells display a higher expression of nutrient transporters when compared to Ly49H^-^ NK cells, indicating the higher metabolic activity of Ly49H^+^ NK cells. Several studies have described a similar phenomenon consistent with our findings. First, signaling through IL-18 and IL-12 is involved in the expansion of Ly49H^+^ NK cells (31). Secondly, during MCMV infection, Ly49H^+^ NK cells display a higher mitochondrial mass that corresponds to their augmented proliferation and expansion to control the viral infection (14, 52, 53). Furthermore, it has been described that direct recognition of infected cells by NK cells, as one of the innate immune sensing mechanism, can in part compensate for MyD88 deficiency (82, 90). Excitingly, the role of Ly49H signaling has recently been observed in adaptive immunity where NK cells with the highest avidity for the m157 antigen of MCMV preferentially multiply to develop into potent memory NK cells with enhanced functionality and protective capacity after MCMV reinfection. Thus, MCMV infection elicits the Ly49H-dependent effects of NK cells (79).

**Figure 7:**
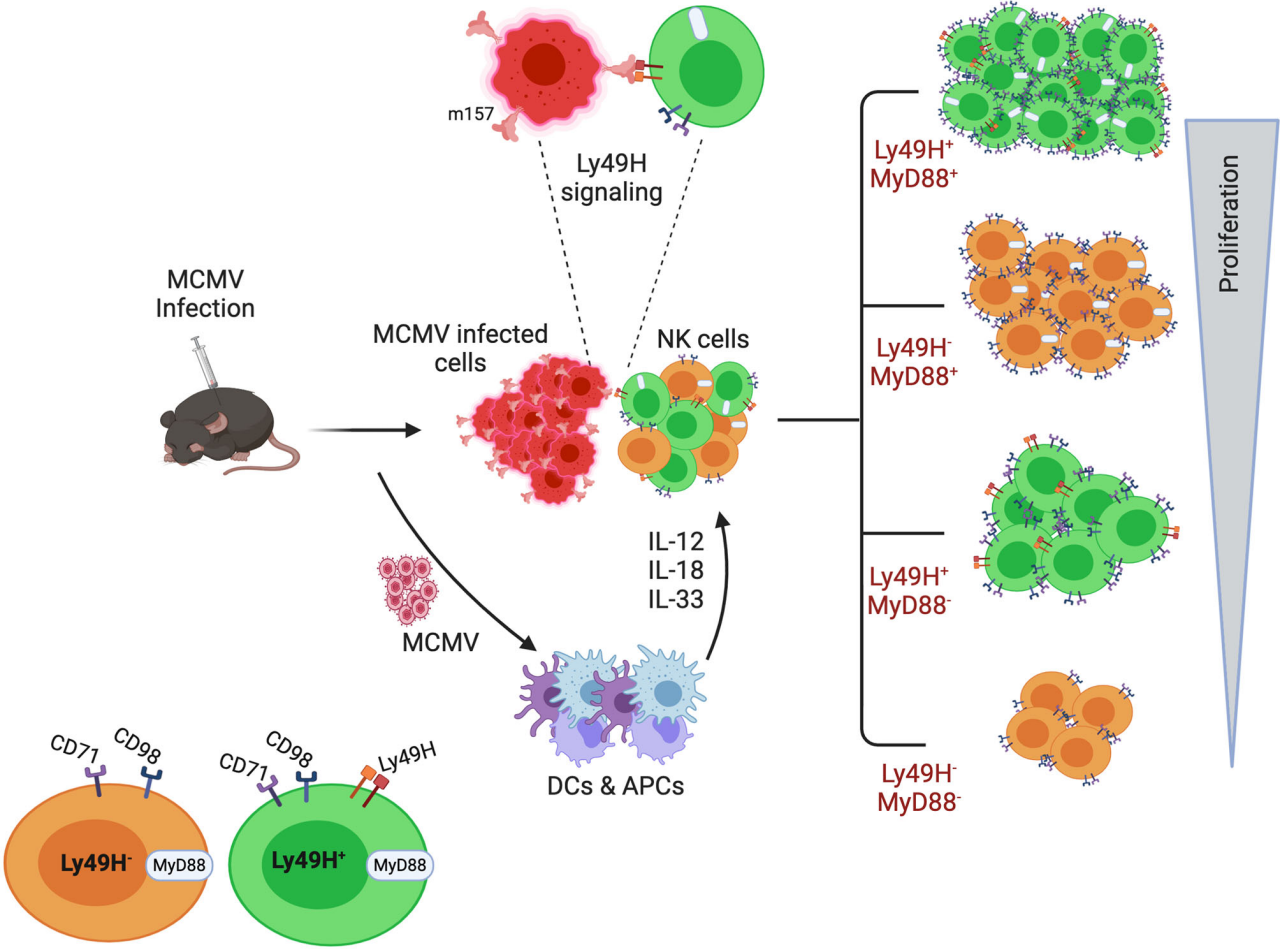
A model for the requirement of upregulated expression of nutrient transporters for the enhanced proliferation of NK cells during MCMV infection. Following infection and inflammation, multiple pathways govern the proliferation of NK cells. Inflammatory cytokines, as well as the activating receptor Ly49H, contribute to the higher metabolic activity of proliferating NK cells. Among the multiple pathways, the MyD88 pathway plays an imperative role in NK cell proliferation. Higher expression of nutrient transporters is required to support the enhanced proliferation of NK cells during MCMV infection.

In summary, we demonstrated that proliferating NK cells have a greater energy demand and exhibit higher metabolic activity, therefore NK cells need to upregulate the surface expression of nutrient transporters. We identified that both cytokines and activating receptors trigger the expression of nutrient transporters on NK cells during infection, resulting in enhanced proliferation of NK cells to control the infection. Furthermore, we described the imperative role of the MyD88 pathway in NK cell metabolic plasticity. In summary, multiple pathways govern the expression of these nutrient transporters that correlates with NK cell proliferation rate (**Figure 7**). This study is particularly significant because the MCMV infection represents the closest homolog of the human CMV (HCMV) infection. Human NKG2C NK cells are considered the counterpart of Ly49H in HCMV, and the cell population is suggested to be adaptive NK cells (91). Several questions remain to be answered; for instance, do other members of the IL-1 cytokine family and other activating ligands play a role in regulating NK cell metabolic activity? Is the failure of nutrient transporter upregulation on NK cells from infected *MyD88^-/-^* mice due to intrinsic MyD88 deficiency, or is it the result of famine production of pro-inflammatory cytokines? What metabolic fuels and signaling pathways are principally used by activated NK cells *in vivo* under limited nutrients? Most importantly, further investigation is warranted for the complete understanding of the molecular mechanisms governing NK cell metabolism during chronic infection. Better understanding will allow researchers to more effectively modulate NK cell function during chronic diseases that involve nutrient-deficient environments, such as cancer.

## Data Availability Statement

The original contributions presented in the study are included in the article/**Supplementary Material**. Further inquiries can be directed to the corresponding author.

## Ethics Statement

The animal study was reviewed and approved by University of Ottawa Animal Care Committee (ACC).

## Author Contributions

AK, SA, and AKA performed the experiments and analyzed the data. AK and SA prepared figures. RS and CAS generated *Il18r1* floxed mice. The original manuscript was written by AK, AKA, and S-HL. AK, LW, and S-HL edited the revised the manuscript. Study supervision was performed by S-HL. All authors contributed to the article and approved the submitted version.

## Funding 

The study was supported by funding from the Canadian Institutes of Health Research (PJT-156106) to S-HL. AK was supported by Mitacs Elevate postdoctoral fellowship program. SA was supported by a scholarship from King Saud University and the Saudi Ministry of High Education, Saudi Arabia, and AKA was a recipient of Ontario Graduate Scholarship Canada.

## Conflict of Interest

The authors declare that the research was conducted in the absence of any commercial or financial relationships that could be construed as a potential conflict of interest.
