# Supplementary material for: Expression of Nutrient Transporters on NK Cells During Murine Cytomegalovirus Infection Is MyD88-Dependent
Source: Front Immunol. 2021 May 3;12:654225. doi: 10.3389/fimmu.2021.654225 (PMC8177011; doi:10.3389/fimmu.2021.654225)
Supplement: Supplementary file 1 [file DataSheet_1.pdf]

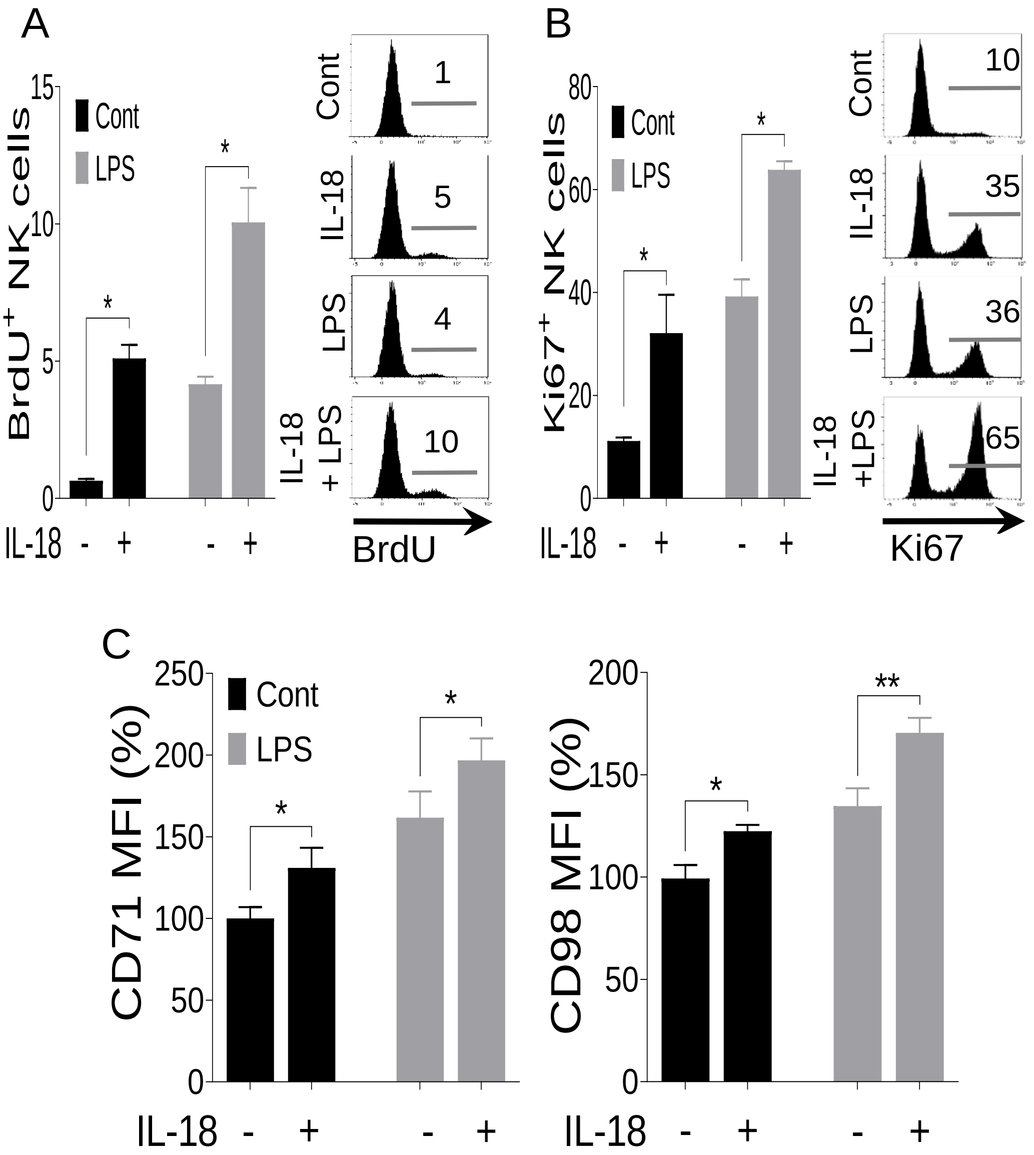

Figure S1. IL-18 signaling promotes NK cells proliferation *in vivo*. C57BL/6 mice were injected intraperitoneally with IL-18 and LPS alone or in combination. Mice were sacrificed at day 2 (D2) post-injection. The percentage of **(A)** BrdU positive and **(B)** Ki67 positive NK cells among total NK in the spleens of mice at D2 post-injection. **(C)** Representative plots depict the mean fluorescence intensity (MFI) of CD71 and CD98 expression on NK cells in the spleen of naive or mice injected with IL-18 and LPS alone or in combination at D2 post-injection. The MFI expression is presented in percentage relative to the MFI of control mice as 100. Data are from one experiment representative of two independent experiments, with three to four mice per group. Data represent mean ± SD. *p < 0.05; **p < 0.01.

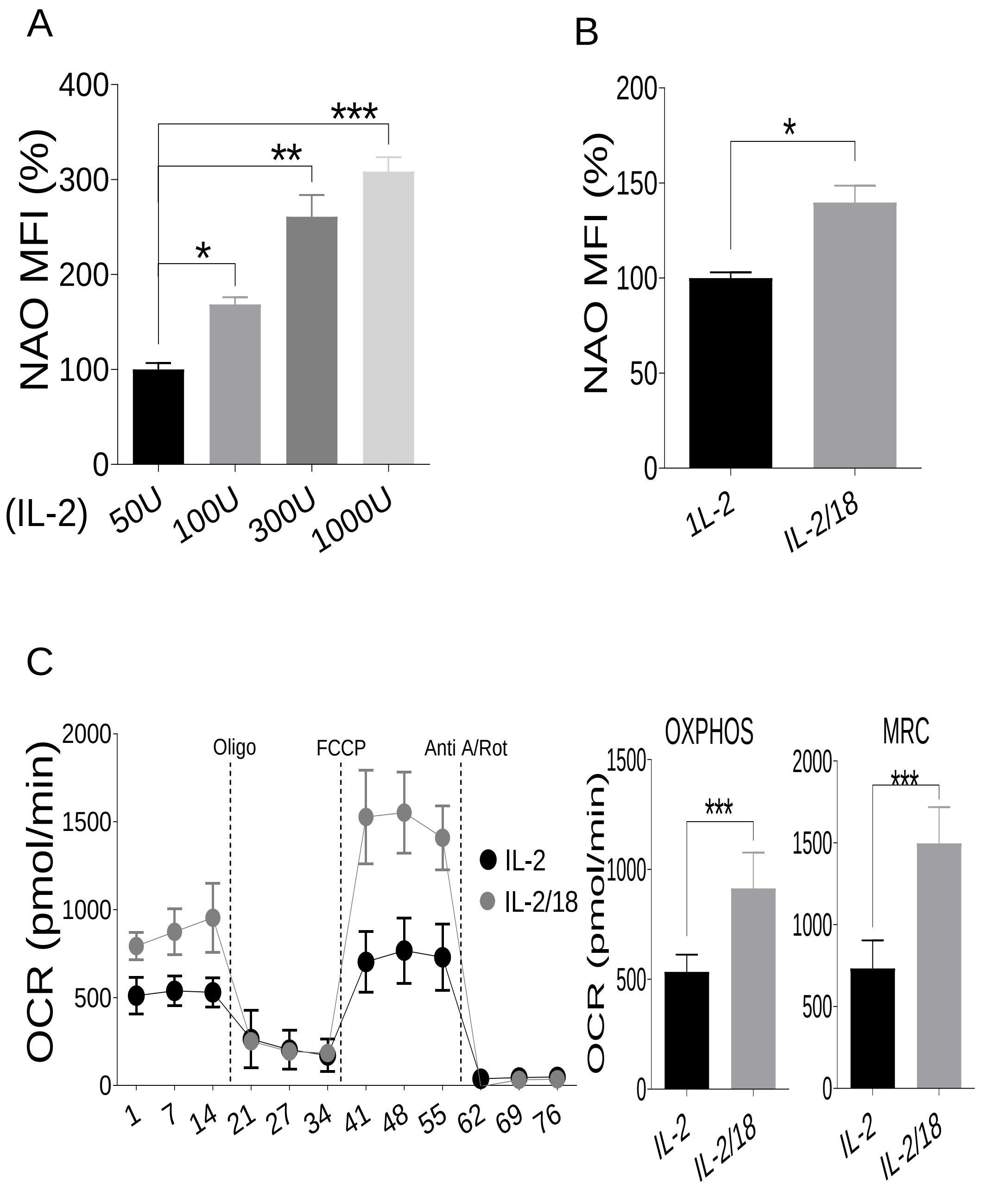

Figure S2. Mitochondrial mass correlates with NK cell proliferation and respiration capacity.

NK cells from spleens of naïve C57BL/6 mice were enriched and **(A)** stimulated with indicated concentration of rhIL-2 for 72 hours or **(B)** IL-18 *ex vivo* for 48 hours where, 100 U/ml of rhIL-2 was added to maintain NK cell survival**.** Representative plots depict the mean fluorescence intensity (MFI) of NAO expression on cytokine stimulated NK cells. **(C)** Analysis of oxygen consumption rate (OCR) of NK cells to asses OXPHOS and maximal respiration capacity (MRC) stimulated with IL-12 or IL2/18 are shown**.** The MFI expression is presented in percentage relative to the MFI of control cells as 100. Statistics are comparing samples to IL-2 stimulated NK cells. Data are from one experiment representative of two independent experiments, with triplicates per group. Data represent mean ± SD.; *p < 0.05; **p < 0.01 ***p < 0.001.

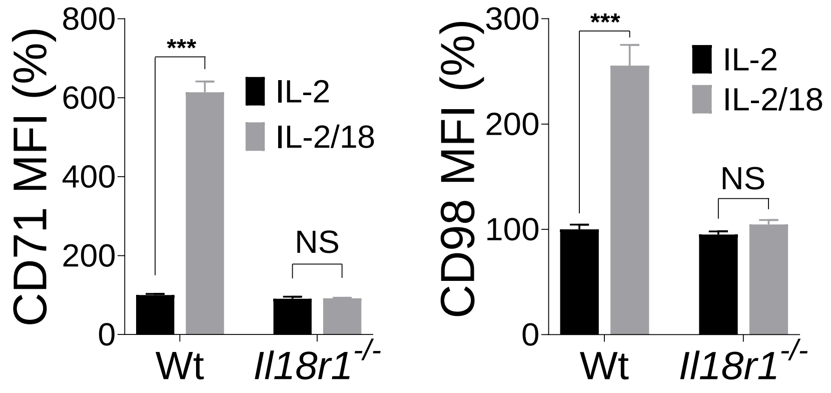

Figure S3. *Ex vivo* IL-18 stimulation did not upregulate nutrient transporters on NK cells from *Il18r1*$^{-/-}$ mice.

NK cells from spleens of naïve C57BL/6 and *Il18r1*$^{-/-}$ mice were enriched and stimulated with the IL-18 *ex vivo* for 24 hours. 100 U/ml of rhIL-2 was added to maintain NK cell survival**.** Representative plots depict the mean fluorescence intensity (MFI) of CD71 and CD98 expression on cytokine stimulated NK cells. The MFI expression is presented in percentage relative to the MFI of unstimulated cells as 100. Statistics are comparing samples to IL-2 stimulated NK cells. Data are from one experiment representative of two independent experiments, with triplicates per group. Data represent mean ± SD. NS, non-significant; ***p < 0.001.

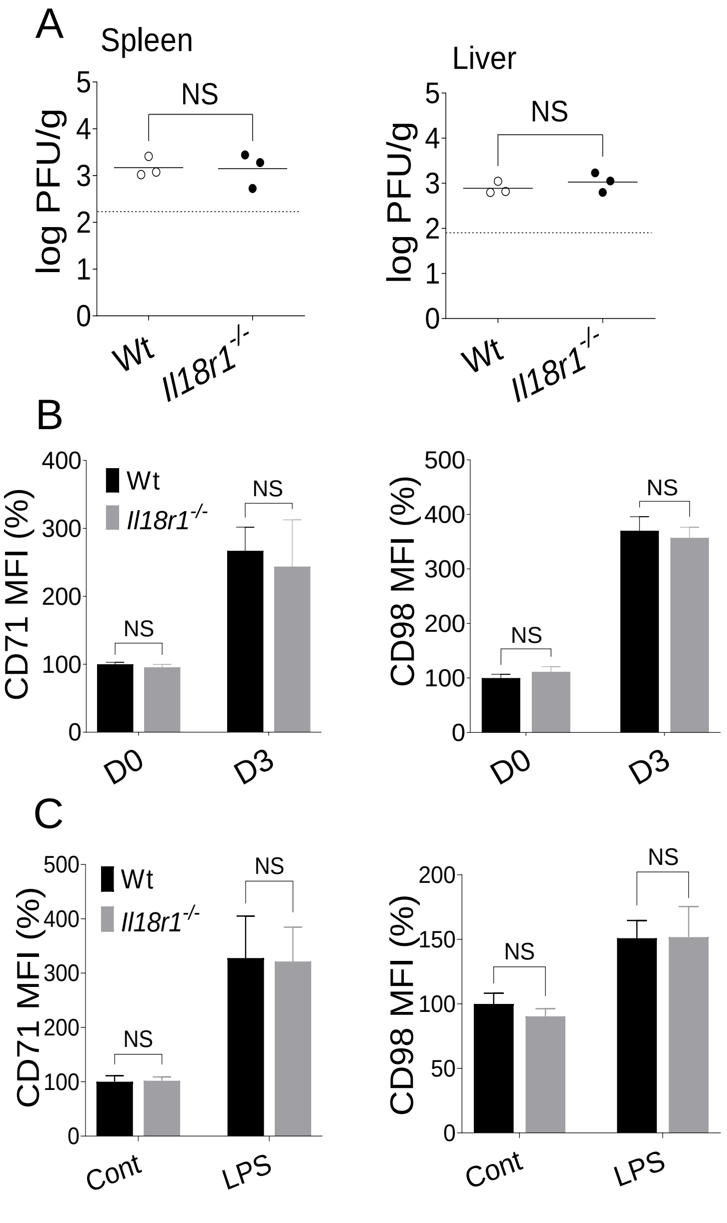

Figure S4. Expression of nutrient transporters on NK cells is independent of IL-18 signaling during MCMV infection.

C57BL/6 and *Il18r1*$^{-/-}$ mice were either left untreated or infected with 3,000 PFU MCMV intraperitoneally and analyzed on the indicated day. **(A)** The viral titers in the spleens and livers of infected C57BL/6 and *Il18r1*$^{-/-}$ mice at day 3 (D3) post-infection. **(B)** Representative plots depict the mean fluorescence intensity (MFI) of CD71 and CD98 expression on NK cells in the spleens of naive (D0) or MCMV-infected mice at D3 post-infection (D3). **(C)** C57BL/6 and *Il18r1*$^{-/-}$ mice were either left untreated or infected with LPS intraperitoneally and analyzed at day 2 (D2) post-infection. Representative plots depict the MFI of CD71 and CD98 expression on NK cells in the spleens of naive or LPS infected mice at D2 post-injection. The MFI expression is presented in percentage relative to the MFI of control mice as 100. Data are from one experiment representative of two independent experiments, with three to four mice per group. Data represent mean ± SD. NS, non-significant.

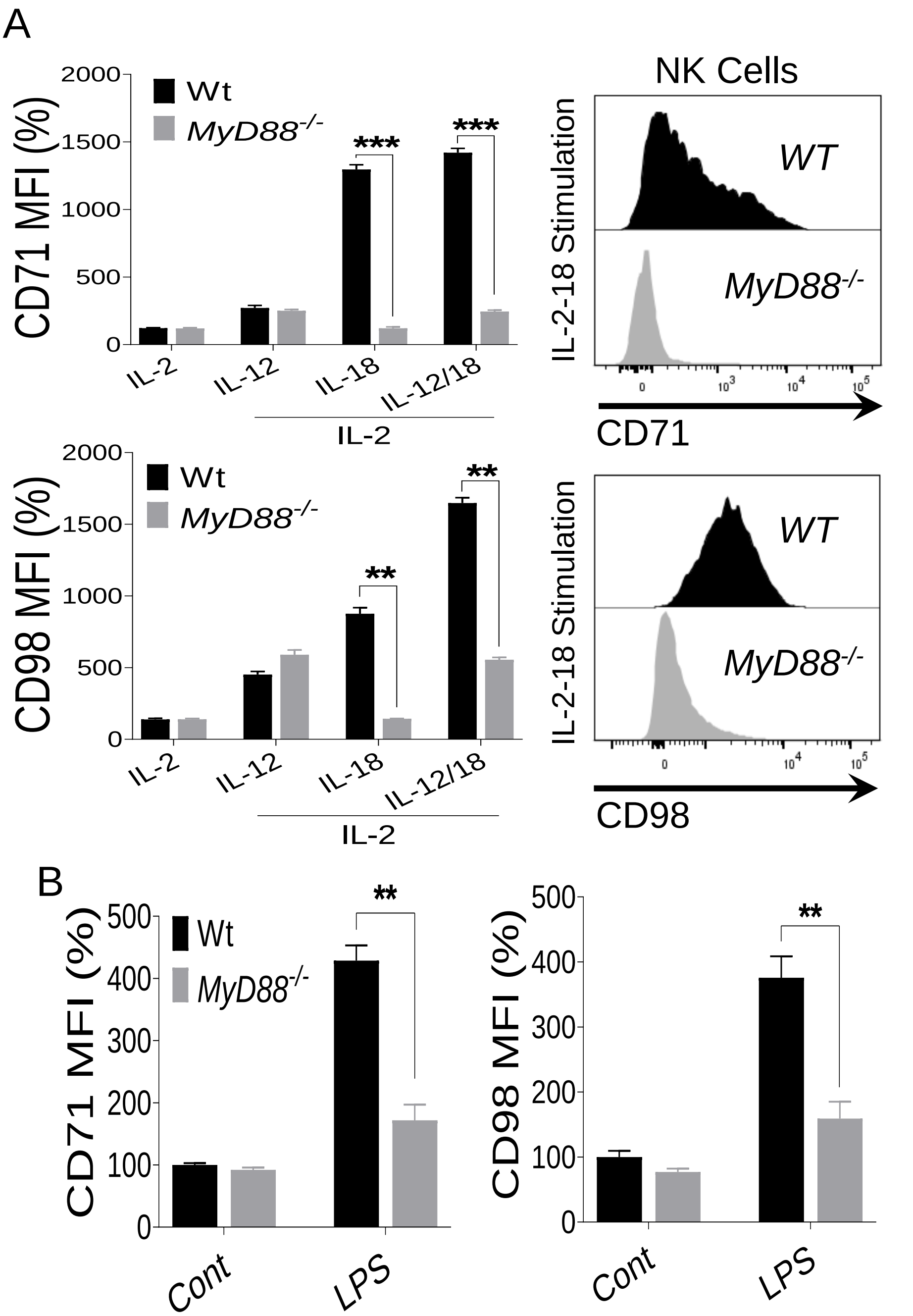

Figure S5. Upregulation of nutrient transporters expression on NK cells is MyD88-dependent.

(**A**) NK cells from the spleens of naive C57BL/6 and *MyD88*$^{-/-}$ mice were enriched and stimulated with the indicated cytokines *ex vivo* for 24 hours. 100 U/ml of rhIL-2 was added to maintain NK cell survival. Representative plots depict the mean fluorescence intensity (MFI) of CD71 and CD98 expression on cytokine stimulated NK cells. Data are from one experiment representative of three independent experiments, with two replicates per group. **(B)** C57BL/6 and *MyD88*$^{-/-}$ mice were either left untreated or infected with LPS intraperitoneally and analyzed at day 2 (D2) post-infection. Representative plots depict the MFI of CD71 and CD98 expression on NK cells in the spleens of naive or LPS infected mice at D2 post-injection. The MFI expression is presented in percentage relative to the MFI of control as 100. Data are from one experiment representative of two independent experiments, with three mice per group. Data represent mean ± SD. **p < 0.01; ***p < 0.001.

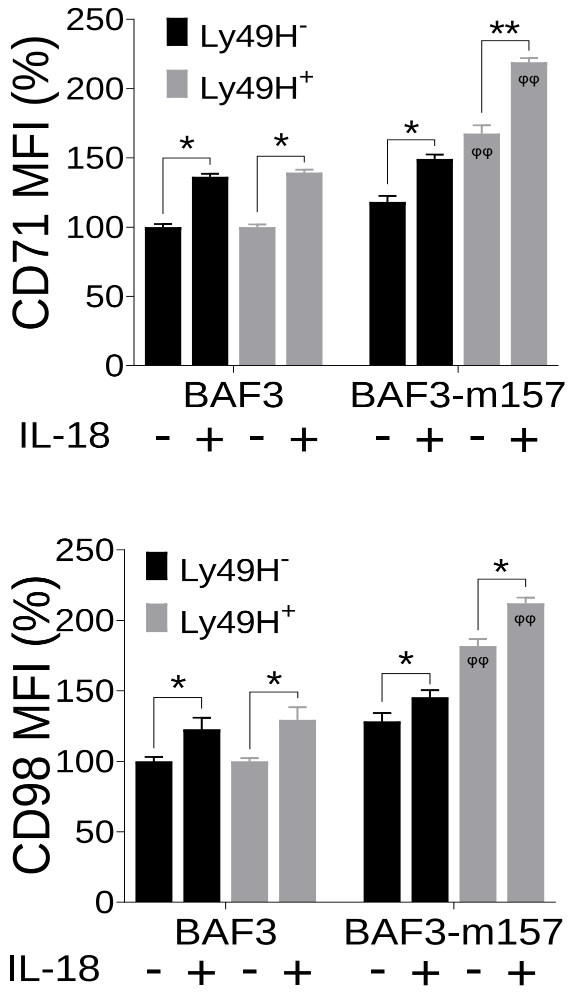

Figure S6. Ly49H signaling is involved in the regulation of nutrient transporters expression on NK cells.

NK cells were enriched from the spleens of naive C57BL/6 and co-cultured with either BAF3 or BAF3-m157 cells in the presence or absence of IL-18 for 18 hours. The mean fluorescence intensity (MFI) of CD71 and CD98 expression on NK cells was measured by flow cytometer. The MFI expression is presented in percentage relative to the MFI of control cells as 100. Data are from one experiment representative of two independent experiments, with two replicates per group. Data represent mean ± SD. $*p < 0.05$; $**p < 0.01$, whereas φ φ $p < 0.01$ statistics are comparing Ly49H+ NK cells incubated with BAF3 or BAF3-m157 cells.
